# Exploring the motivations of patients with type 2 diabetes to participate in clinical trials: a qualitative analysis

**DOI:** 10.1186/s40900-016-0050-y

**Published:** 2016-12-12

**Authors:** Stephanie Estcourt, Jill Epton, Tom Epton, Bijay Vaidya, Mark Daly

**Affiliations:** 1 Department of Research & Development, Royal Devon & Exeter Hospital, G9 Child Health Building, Barrack Road, Exeter, EX2 5DW UK; 2 Patient Public Involvement Group Representatives, Exeter, UK; 3 MacLeod Diabetes & Endocrine Centre, Royal Devon & Exeter Hospital, Exeter, UK

**Keywords:** Research, Diabetes, Motivations, Patient participation

## Abstract

**Plain English Summary:**

Certain patient groups are reluctant to engage with clinical research and consequently findings are not always truly representative of the wider population. With the emphasis on evidence-based clinical practice, clinical research as a core activity for the National Health Service (NHS) and the rising prevalence of diabetes within the United Kingdom (UK) it is important to understand what motivates people to volunteer for research in diabetes and identify the barriers to this involvement. This research interviewed 12 people with type 2 diabetes who had previously taken part in diabetes clinical trials. The transcripts of these interviews were analysed to identify themes that informed the study findings.

There were wide ranging reasons for participating in clinical trials. Both altruistic and self-interest motivation were universally expressed. The thought of helping others was a powerful experience but for some there was a sense of duty to volunteer especially if they had benefited from NHS care. Participating was empowering, with extra access to healthcare professionals, practical information and support for their condition. Coping with the logistics of being in a trial relied upon a strong network of family and friends. Some felt anxious at the end of the trial having been supported during the research and appreciating the camaraderie of belonging to a group or team.

This study provides insights into the motivations and barriers to involvement in clinical research in type 2 diabetes helping researchers to encourage and support more volunteers in clinical trials.

**Abstract:**

**Background** Certain patient groups are reluctant to engage with clinical research and consequently findings of the research are not always truly representative of the wider population. This, together with a growing prominence of evidence-based clinical practice, an increasing emphasis of clinical research as a core activity for the NHS and the rising prevalence of diabetes within the UK population, requires an understanding of motivations and barriers for patients consenting to participate in diabetes clinical trials.

**Methods** To understand patients’ motivations for participating in clinical trials in type 2 diabetes. We conducted a qualitative study involving 12 participants with type 2 diabetes with previous involvement in clinical trials. Individual, tape-recorded, semi structured interviews were conducted to explore motivations and experiences of the participants. We carried out thematic content analysis to identify themes, from which theoretical interpretations were formed.

**Results** There were wide ranging reasons for participating in clinical trials. We identified 3 key themes: (1) Motivations ranged from altruism to self-interest; (2) participation in clinical trials was an empowering experience; and (3) key to participation was a strong network of support.

**Conclusion** Patients are motivated to participate in clinical trials by a sense of altruism coupled with self-interest. This self-interest centres on the belief that participation would be an informative and empowering experience with increased access to healthcare professionals. However the ability to cope with the logistics of being in clinical trials relies upon an extensive and reliable network of support from family, friends, work colleagues and employers, together with a collaborative approach to their care from the researchers and their usual healthcare providers.

## Background

The prevalence of diabetes is increasing with over four million people in the United Kingdom diagnosed with diabetes representing an increase of 65% during the last decade [1]. As the number of people living with diabetes increases there is an increased burden on the National Health Service (NHS). Recent national audits revealed that only 60% of people with diabetes get the eight health checks recommended by the National Institute for Health and Care Excellence (NICE) and even less are offered structured education on managing their disease [2]. Large numbers of patients will end up experiencing potentially preventable diabetes-related complications such as blindness, kidney failure and amputation. Complications related to the diseases account for a substantial proportion of the direct health costs. As the prevalence of diabetes increases, the cost of treating its complications will grow if current care regimes are maintained [3].

There are increasing calls from patient groups, the major charitable groups that fund research, the Department of Health (DOH) and the National Institute of Health Research (NIHR) to invest in research that looks at potential cures, treatment and prevention of diabetes. Together with an emphasis on enabling patients and service users of the NHS to engage with the research process, the need to recruit participants to clinical research has never been more crucial. The NHS Constitution identifies research as a core activity making extensive reference to the significance of conducting research, using research evidence in clinical decision-making and the crucial importance of patient involvement [4].

Clinical trials providing evidence for all aspects of diabetes prevention, diagnosis and care rely on successful recruitment to clinical trials.

It is recognised that there are groups of patients reluctant or unable to participate in clinical trials in other healthcare areas [5, 6] whilst there is limited evidence to indicate what motivates people with type 2 diabetes to accept or decline participation in clinical trials. In 1998, Verheggen et al. [7] suggested what motivates participation in clinical trials is a considered evaluation of the pros and cons of taking part. More recent studies in patients with spinal cord injuries [8] and angina management [9] identified the need for healthcare professionals (HCPs) to generate systems encouraging research involvement. A Canadian paper investigating perceptions of participation in cardiovascular clinical trials identified altruistic motivation for involvement as well as a desire for more information from the participants [10]. Whilst the experience of research involvement for a group of patients with irritable bowel syndrome included identifying the desire for a thank you note, summary of trial results at the end of the study and monetary recompense for expenses [11]. Although the literature searches identified studies that explored patient motives and barriers for involvement in research this predominantly related to clinical trial involvement and did not explore how patients felt at the end of the experience.

The main body of evidence for exploring patients’ experiences of involvement in research can be found in the field of cancer. There is clear evidence for why patients refuse to join randomised trials [12] including fear of the research, fear of the illness, a desire to have ‘tried and tested treatment’, and their motivations and barriers to involvement [13, 14]. One qualitative study looking at cancer patients’ experiences whilst in a trial concluded that contact with and support from the research nurse was an important and valued feature of participating in a trial [15]. However no studies exploring the research experience of patients with diabetes were found. Therefore we have carried out a qualitative study with the aim of investigating patients’ motivations for participating in diabetes clinical trials in order to identify and understand potential barriers to recruitment.

## Methods

We conducted hour long semi-structured interviews with 12 participants with type 2 diabetes. A total of 29 patients were approached for the study, 10 declined due to time constraints and seven because of a reluctance to be interviewed. Of the participants, eight were male subjects; median age was 64 years (range 48 – 78 years), duration of diabetes ranged from 3 to 25 years. All participants had been involved in randomised controlled trials within the last 2 years. Six had taken part in more than one trial and a further three had failed to complete the research.

At the initial stages of protocol development two lay people with type 2 diabetes were identified from the Public Patient Involvement (PPI) group. Their role was to help develop the protocol, documentation and comment on the findings.

Interviews were conducted either in the hospital or at participants’ homes using open-ended, neutral questions that related to previous research experiences ensuring that key areas were impartially and consistently explored (Appendix). The interviewer was not known to the participants. Standard audiotaping and transcription methods were used. We performed thematic content analysis to identify key themes and issues, using a system of indexing the printed transcripts [16] which provided descriptions of both common and unique experiences. Data saturation would not have been possible with the available population that was suitable in terms of inclusion criteria, however there was a sense of commonality of experience from the 12 participants. Emerging themes were verified by both the researchers and lay patient representatives. This PPI aspect to analysis enhanced validity by reducing HCP bias and providing contextualisation for the emerging themes. An on-going process of analysis identified core categories that were explored in subsequent interviews [17]. Preliminary results were posted to all participants who then had the opportunity to feedback on the key themes and issues identified. This process of negotiated feedback enhances validity and reduces the potential for researcher bias. Final analysis developed specific categories from which theoretical interpretations were formed.

## Results

Three key themes were identified: (1) altruistic motivation and self-interest; (2) personal empowerment as a result of being involved; and (3) adapting to cope with the research process.

### Motivation

There were wide ranging reasons that had prompted participation in clinical trials and these motivations were influenced by previous life experiences. Two categories of motivation were identified; altruistic and self-interest.

#### Altruistic motivation

The concept that their participation in research would potentially help others was expressed in similar terms by most participants and viewed as a positive experience.

*‘My idea was anything that is going to help anyone in the future with it (diabetes), if I can help, yes please.’*_(07: 80–82)_

For others this sense of altruism was expressed as a sense of duty to volunteer and to set a good example to others.

*‘If not enough people come forward, nobody’s going to know.’*_(08: 19–21)_

This sense of duty was also expressed in the form of giving something back whilst investing in the future for the benefit of others.

*‘You give something back and if it helps find a cure in the future, it might not find a cure for me, which I don’t think it will, but in the future it might help further down the line and that was my reason for doing it.’*_(02: 86–89)_

Many participants felt a sense of achievement at having engaged with the research process whether they completed the study or not and would be happy to be involved again.

#### Motivation of self interest

Self-interest was the universal and overwhelming motivation for this group.

*‘The main reason is really a selfish reason; I hoped it would help me.’*_(10: 75)_

There was a quest for knowledge regardless of duration of diabetes and being involved in research was seen as a legitimate way of accessing information and addressing individual concerns about health or treatment.

*‘I thought at least I would get some useful information because every time I went to a different doctor or nurse at* (the) *surgery, they would tell you something different.’*_(03: 154–156)_

Involvement in research was also viewed as a way of gaining extra access to healthcare professionals.

*‘I was getting regular medical check-ups so I was quite happy with that, I was being kept an eye on from a diabetic point of view.’*_(04: 84–85)_

It was generally accepted that access to primary healthcare professionals was time limited, whereas this was not always the case with the research team who had both the time and expertise to provide support and education.

*‘Your GP only gives you 10 min, it’s general, it’s not as intensive as this study which is purely for diabetes.’*_(07: 101–2)_

Seeking information was a spur for those with a strong family history of diabetes.

*‘I wanted more knowledge….my son-in-law is diabetic and I wanted to know if any of my grandchildren would get it.’*_(05: 51)_

*‘It’s highly probable my two daughters will become diabetics and if I can do anything to help them it would be a great thing to do.’*_(06: 8–9)_

Motivation was closely associated with current health issues; managing blood sugars, controlling diet and the efficacy of current treatments.

*‘There was the other reason why I was really interested in the trial, because I didn’t want to have to go on insulin.’*_(10: 148–9)_

*‘I did it* (the research) *because I wasn’t feeling 100%.’*_(07: 86)_

‘*I have always struggled with my diabetes, I could never lose weight.’*_(08: 86)_

Whilst one participant stated that they had declined involvement because they felt their diabetes was well controlled and they were unwilling to compromise that. For some, clashes of work and personal life with the study schedules precluded their involvement.

*‘It was taking too much of the (employer’s) time when I felt that my records showed that I was looking after my diabetes, I was controlling it, so I didn’t think I needed to do so.’*_(01: 133–5)_

Experience of research motivated 6 of the participants to be involved in more than one study and others happy to consider future involvement.

### Personal empowerment

Empowerment resulted out of gaining greater knowledge and insight into diabetes, its care and treatment. This was achieved through educational, practical and psychological support both from the research healthcare professionals, and the participants’ family, friends and workplace.

#### Educational support

The research process enabled participants to gain a greater knowledge of their diabetes regardless of the duration of their diagnosis.

*‘I’ve learnt a lot....that diabetes is a progressive illness and I couldn’t get my head around that….so it* (the research) *helped me in that way to understand what diabetes was.’*_(04: 197–207)_

*‘I learnt quite a few things, I have been diabetic for quite a few years and I was thinking ‘I didn’t know that!”*_(08:0234–5)_

The results showed that education had a positive impact on perceived health outcomes, and that provision of information resulted in a clearer understanding of diabetes and related issues.

*‘I’m now back within the normal confines of sugar levels.’*_(02; 157)_

However the lack of information or feedback about the research itself was extremely frustrating for several participants. There was a general sense of disappointment about the lack of information at the end of the study and no sense that feedback about results had been received.

*‘I would have liked to have known what was the outcome of the trial.’*_(05: 235)_

#### Practical support

Practical support enabled participants to feel empowered to make changes to aspects of their daily life such as dietary choices and cooking habits. Some participants even felt confident enough to make practical differences to diabetic control as a result of taking part in research.

*‘Like bring my insulin down to match my food intake. I would not have had a clue how to do that before, I would not have thought about it.’*_(03: 170–1)_

This practical empowerment translated into quantifiable measures such as weight loss, less hypoglycaemic episodes, better blood glucose and glycated haemoglobin (HbA_1_C) readings. Participants gained practical skills including accurate and appropriate recording of blood sugars, how to inject insulin and manage hypoglycaemic events.

*‘During the trial I ended up losing 2½ stone and also my diabetic HbA*_*1*_ *C dropped it to where they like it, what I call normal.’*_(08: 221–3)_

#### Psychological support

Psychological support from a variety of sources enabled participants to become and remain involved with research and complying with the research specific activities by absorbing them into their daily routine.

*‘It’s not difficult to achieve really and at the end of the day if you add up all the time that it takes…it’s probably less than 5 min, so it’s not particularly onerous, it’s just a question of fitting it in.’*_(11: 319–22)_

Participants felt as if they were cared for by the research team in a supportive and collaborative environment.

*‘The nurses work with you, rather than you are the patient.’*_(04: 280)_

Feeling supported whilst learning new techniques such as injecting, enabled participants to cope with their diabetes.

*‘It’s the same with any illness, you don’t want it, the ‘why me’, I know it isn’t life threatening but I didn’t want it and I didn’t want to come to terms with it, but she* (the research nurse) *helped me in that respect.’*_(07: 135–7)_

The support from family and friends was, in some cases, pivotal to some participants joining a research study.

*‘We knew it* (the research schedule) *would restrict his life as well as mine so that is why we discussed it.’*_(05: 80–2)_

Likewise, having a supportive employer in order to negotiate the time in which to attend research clinics was important to some participants. Although combining a research schedule with work could become a barrier to joining further research.

*‘They invited me up for further research a few years later, I declined because I couldn’t take any further time off from my employment.’*_(01: 76–8)_

The relationship built up with the research team created an environment that made participation possible and enjoyable. Easy access to the research team was also appreciated and regarded as beneficial and reassuring.

*‘It wasn’t like going to see a doctor and a nurse, it was almost like going to see 2 friends and I think that was exceptional, because that actually makes it so much easier.’*_(06: 79–81)_

However, the end of a study caused anxiety associated with no longer having increased and easy access to healthcare professionals.

*‘You are sort of in this cocoon really and suddenly you are cast aside you know, we have finished with you know, off you go!’*_(09: 246–9)_

## Coping with the research process

Logistical problems coping with the research visits were commonly reported. These repeatedly involved access to the hospital site with concerns about parking issues.

*‘I had a problem when I first came up, parking.’*_(02; 179)_

There seemed to be an expectation that this was the ‘norm’ and had been a common experience when attending clinical outpatient appointments and, although noted by participants, did not stop them from joining research studies. Study schedules especially those that required early morning fasting proved problematic for some.

*‘I had a lift with a friend, because I was fasting, live on my own, had I not had a friend to bring me up in the morning, it might have posed a little bit of a problem.’*_(07: 238–43)_

Logistical issues were generally easy to cope with but for many the prospect of finishing the research and the anxiety associated with going back to their usual healthcare providers was difficult to cope with. Many seemed surprised that they had felt this way especially after feeling so supported during the research and having appreciated the camaraderie of belonging to a group or team.

*‘You can talk to other people who are in the study and you get to know what other people’s diabetes is like.’*_(04; 202–3)_

On occasions participants were aware of conflict between the research and primary care teams, which served to heighten anxiety at the point of discharge. Several discussed the difficulties associated with continuing with treatment initiated by the research team

*‘Unfortunately we can explain to him* (GP) *that it* (the study drug) *has helped you, so it’s in his best interest anyway, but he doesn’t have to prescribe it.’*_(10: 243–5)_

*‘I think they went to a lot of trouble afterwards, because I think that my GP was dragged kicking and screaming into providing me with [the medication] and I think letters were written otherwise I’m sure I wouldn’t have got it.’*_(12: 288–90)_

By facilitating a smooth hand over to the primary care teams, the researchers were able to help ease these feelings and help participants to cope with the transition.

*‘They informed my GP all the time of what was going on and he was quite happy and come the end because the insulin and [medication] worked so well and brought down my HbA*_*1*_ *C down to a normal level they decided to keep me on it.’*_(08:273–77)_

## Discussion

This qualitative study of patients’ experiences participating in diabetes clinical trials provides insight into the motivations, feelings of empowerment and the use of coping strategies whilst engaged with the research process. It is recognised that qualitative methodology is the most appropriate means of capturing this type of experiences [18]. Due to the small numbers our findings provide insight into patient’s motivation for engagement with clinical trials is both altruistic and influenced by self-interest. The ability to cope whilst participating relies upon a clear support network including family, friends, work colleagues and employers, together with collaborative care from the researchers and patients’ usual healthcare providers.

This study has several limitations. Although more than 15,000 people participate annually in clinical trials in different specialities in the South West Peninsula, we explored the experiences of a small cohort of participants in diabetes research in this study. The study population was also limited to those from rural communities and did not include people from minor ethnic groups or people who had declined participation in clinical trials. They may have identified different issues for not participating or engaging with research. However, a purposive or judgemental sample method was used in this study to ensure that participants with type 2 diabetes who had been recently consented onto a clinical trial were selected. Combining purposive with maximum variation sampling ensured that a range of perspectives including gender, age, social class, family structure and disease status, were captured. The themes identified in this study resonate with a range of research from a wider perspective of diseases and research methodologies and add to the specific body of knowledge surrounding participation in diabetes research. A major of strength of this study is the involvement of PPI representation at all stages, from protocol development to commenting on the results and contributing to the preparation of the manuscript.

Both PPI representatives volunteered to be involved in the development of research. Neither came from a healthcare background but had an understanding of research and diabetes. Their roles were to provide a critical eye on the research, questioning researcher roles and assumptions, whilst sharing their personal understanding and reflections on their experiences of research involvement [19]. Their involvement ensured that the research process focused on what was important to the participants [20]. This is identified as a pivotal role for PPI involvement in forming a *research ethos* and subsequently shaping appropriate research questions and results [21]. Recent Health Research Authority and INVOLVE briefing and guidance documents endorse this, indicating that research that involving the public is more likely to be relevant and of a higher quality [22].

Investigations of patients’ motives for participating in clinical trials have been carried out in a variety of healthcare settings. In 1998, a survey of participants of 26 clinical trials found that patients base their decision to join a study by making a *personal balance account,* by which they weigh up the pros and cons of participation by looking for some added physical or emotional gain whilst taking into account the potential risks and extra time that will be involved [7]. Participants in our study confirmed this process of deliberation: being recruited into a clinical trial ensured greater access to healthcare professionals, education about their diabetes and the potential for more personalised care and treatment.

Our study indicates that the concept of self-interest is a powerful motivation and feedback from the participant verification process confirmed that this was an intrinsically personal motivation whilst altruism reflected a more selfless desire to *‘do good’* or *‘help others’*. Information and communication about participation in clinical trials is a balancing act for healthcare professionals, between providing sufficient information about the potential risks and benefits in order to facilitate enrolment whilst facilitating patients’ informed decision making [13, 14]. Our study revealed some interesting insights into why this discrete cohort of patients decline involvement at other stages in their disease. Feeling well and in control of one’s diabetes reduces the motivation of self-interest and the idea of altruism is not a great enough motivation to participate. This may be difficult to address within the research teams because the consent process involves the explicit discussion that there may be no benefit to involvement. This stems from the need to ensure that, as in the case of studies using placebo, patients understand that there may be no physical/clinical benefit. However, the power of the placebo effect [23, 24] and the enhanced clinical outcomes for patient involved in research whether on placebo or not, are well noted [25–27].

The concept of personal benefit whilst helping others is also echoed in multinational studies across a range of disease areas [28]. A study exploring the motivations of parents for enrolling their children onto clinical trials identified that their decision for enrolment was guided by possible benefit to their own children and the potential for benefiting other children in the future [29]. This combination of altruism combined with the motive of gaining some benefit is a recognised phenomenon [10], whilst other identified incentives for participation included the use of thank you notes and summary of the trial results [11].

Our study identified personal benefits associated with participation in clinical trials, with many of the participants expressing a sense of empowerment as a result of taking part. And whilst it was a small group that was interviewed, their experiences resonate within the wider context of current research into motivations. This process of empowerment was multifaceted and achieved through educational and practical support from the research team and psychological support from partners, family and crucially from the workplace for those in employment.

It is clear that patients’ expectations of routine care are discordant with current clinical provision. This raises questions about what elements are missing from the clinical consultations that many of this group of patients will routinely experience throughout the year. What is it that they are seeking from the extra contacts experienced as part of a research schedule? Many of the participants reported that they were seeking further clarification and support through joining a clinical trial because they felt that these needs were not being met by their usual healthcare providers. In keeping with our results, studies exploring the needs of patients participating in cancer clinical trials concluded that contact with, and support from the research nurse was an important and valued feature [15]. This together with a sense of bereavement at the end of trial participation [30] may explain why several patients had participated on more than one trial.

This study also confirms a range of barriers related to the practicalities of participating in clinical trials for this small cohort of patients. There are inherent difficulties in trying to combine the schedules and requirements for research with employment and family commitments. Physically accessing the research service was acknowledged as difficult and usually referred to the limitations of car parking, inappropriate public transport schedules, and the barriers to the use of bus passes. These findings corroborate earlier studies predominantly in the field of cancer research [12, 31, 32], and during the last decade research has consistently identified the need for healthcare professionals to generate a research service that meets the needs of those patients who want to participate in research [8, 9]. Whilst it is recognised that clinical trials are crucial in the development, evaluation and progression of health and social care, recruitment into clinical studies remains historically low and continues to be a challenge for researchers [33].

## Conclusion

Whilst this study interviewed a small cohort of patients, their experiences replicate those identified by studies into participants’ motivations across a wider spectrum of clinical and research settings. This study provides insight into the motivations of a small cohort of patients participating in diabetes clinical trials confirming that they were motivated by a sense of altruism coupled with self-interest. They believed that participation in diabetes research was an informative and empowering experience. The ability to cope with the logistics of taking part in research relied upon clear support network including family, friends, work colleagues and employers, together with collaborative care from the researchers and the patients’ usual healthcare providers.

## Appendix

**Interview Guide** Version1, 15.06.2011

*These questions have been discussed and verified with the users, their feedback on the questions, their appropriateness, relevance and order has influenced the interview guide*

- “Tell me about yourself” *(age, family and social background)*
- “What sort of research have you been involved with” *(trials, research databases, observational studies, duration, level of commitment, visit schedule)*
- “What made you want to volunteer for the research” *(allows discussion and exploration of individual’s motivations, altruism, hoping to gain from it)*
- “What impact did your involvement in research have on your everyday life” *(other commitments, work, social life, others responses, partners, family and friends)*
- “Why did you get involved” *(influences, choices, motivations)*
- “Tell me how you coped with being involved in the research” *(what adjustments did they make, how did they accommodate the experience, coping strategies)*
- “What sort of information were you given prior, during and at the end of the research” *(what explanations they were given, were they involved in decision-making, their understanding and levels of satisfaction with their care)*
- “How do you feel about your experience of being involved in research” *(emotional reactions)*
- “What additional support would you have liked” *(what would have helped then and now)*
- “Are there any other issues you would like to raise?” *(allows the participant to raise issues that may have been missed by the researcher and users)*

## Abbreviations

DOH: Department of health
HCP: Healthcare professional
NHS: National Health Service
NICE: National Institute for Health and Care Excellence
NIHR: National Institute for Health Research
PPI: Patient Public Involvement
UK: United Kingdom
